# Assessment of the Immune Response in Patients with Insulin Resistance, Obesity, and Diabetes to COVID-19 Vaccination

**DOI:** 10.3390/vaccines11071203

**Published:** 2023-07-05

**Authors:** Jędrzej Warpechowski, Paula Leszczyńska, Dominika Juchnicka, Adam Olichwier, Łukasz Szczerbiński, Adam Jacek Krętowski

**Affiliations:** 1 Clinical Research Centre, Medical University of Bialystok, Sklodowskiej-Curie 24A, 15-276 Bialystok, Poland; pleszczynska1@student.umb.edu.pl (P.L.); djuchnicka1@student.umb.edu.pl (D.J.); aolichwier2@unl.edu (A.O.); lukasz.szczerbinski@umb.edu.pl (Ł.S.); adamkretowski@wp.pl (A.J.K.); 2 Department of Nutrition and Health Sciences, University of Nebraska–Lincoln, Lincoln, NE 68588, USA; 3 Department of Endocrinology, Diabetology and Internal Diseases, Medical University of Bialystok, Sklodowskiej-Curie 24A, 15-276 Bialystok, Poland; 4 Center for Genomic Medicine, Massachusetts General Hospital, 185 Cambridge Street, Boston, MA 02114, USA; 5 Programs in Metabolism and Medical and Population Genetics, Broad Institute of MIT and Harvard, 75 Ames Street, Cambridge, MA 02142, USA

**Keywords:** diabetes mellitus type 2, diabetes mellitus type 1, obesity, BNT 162 vaccine, 2019-nCoV vaccine mRNA-1273, Ad26COVS1, ChAdOx1 nCoV-19, SARS-CoV-2, immunogenicity, vaccine

## Abstract

The SARS-CoV-19 pandemic overwhelmed multiple healthcare systems across the world. Patients with underlying medical conditions such as obesity or diabetes were particularly vulnerable, had more severe symptoms, and were more frequently hospitalized. To date, there have been many studies on the severity of SARS-CoV-2 in patients with metabolic disorders, but data on the efficiency of vaccines against COVID-19 are still limited. This paper aims to provide a comprehensive overview of the effectiveness of COVID-19 vaccines in individuals with diabetes, insulin resistance, and obesity. A comparison is made between the immune response after vaccination in patients with and without metabolic comorbidities. Additionally, an attempt is made to highlight the mechanisms of immune stimulation affected by SARS-CoV-2 vaccines and how metabolic comorbidities modulate these mechanisms. The focus is on the most common COVID-19 vaccines, which include mRNA vaccines such as Pfizer-BioNTech and Moderna, as well as viral vector vaccines such as AstraZeneca and Johnson & Johnson. Furthermore, an effort is made to clarify how the functional differences between these vaccines may impact the response in individuals with metabolic disorders, drawing from available experimental data. This review summarizes the current knowledge regarding the post-vaccination response to COVID-19 in the context of metabolic comorbidities such as diabetes, insulin resistance, and obesity.

## 1. Introduction

The first case of severe acute respiratory syndrome coronavirus 2 (SARS-CoV-2) was reported in December 2019, and the outbreak was identified in Wuhan City in China [1]. The virus quickly spread worldwide, causing a pandemic [2]. SARS-CoV-2 can be directly transmitted from one person to another through the droplet route, mainly during sneezing and coughing, but also through speaking [3]. Another path of spread is indirect contact, such as being exhaled by an infected person, virus transmission into mucous membranes of the eye, mouth, or nose after touching surfaces or objects contaminated with the virus [4]. What is extremely important is the virus spreads more easily in closed and crowded spaces [4].

Betacoronaviruses, including SARS-CoV-2, cause respiratory or gastrointestinal diseases in humans [5]. They can cause mild colds and severe pneumonia [6]. The main symptoms of the mild form of the disease include high fever, dry cough, fatigue, sore throat, muscle pains, nasal obstruction, sneezing less often, loss of taste/skiff (which may represent the only symptom of the disease), diarrhea, or rhinorrhea [7,8].

SARS-CoV-2 belongs to the large family *Coronaviridae* [2]. Coronaviruses are enveloped viruses, and their genome consists of single-stranded, positive-sense RNA [9] with core transcriptional regulatory sequences 5′-CUAAAC-3′ or 5′-CUAAAC-3′ [10]. The genetic material of SARS-CoV-2 consists of 14 open reading frames (ORFs) [11], which encode essential proteins, both structural, non-structural, and accessory [11]. One of the most critical proteins is spike glycoprotein (S protein), which belongs to the group of structural proteins [12]. S protein forms spikes, which play a role in the binding and entry of the virus into the host cell by attaching to the angiotensin-converting enzyme 2 (ACE2) receptor on the cell surface of respiratory epithelial cells [12]. ACE-2 receptors mainly use spikes in the lung epithelial cells [12]. Non-structural proteins play a critical role in viral RNA replication and defense against the host immune system, while accessory proteins act as intermediaries, participating in transport in host cells; they help in viral infection and virus survival [13].

**Figure 1:**
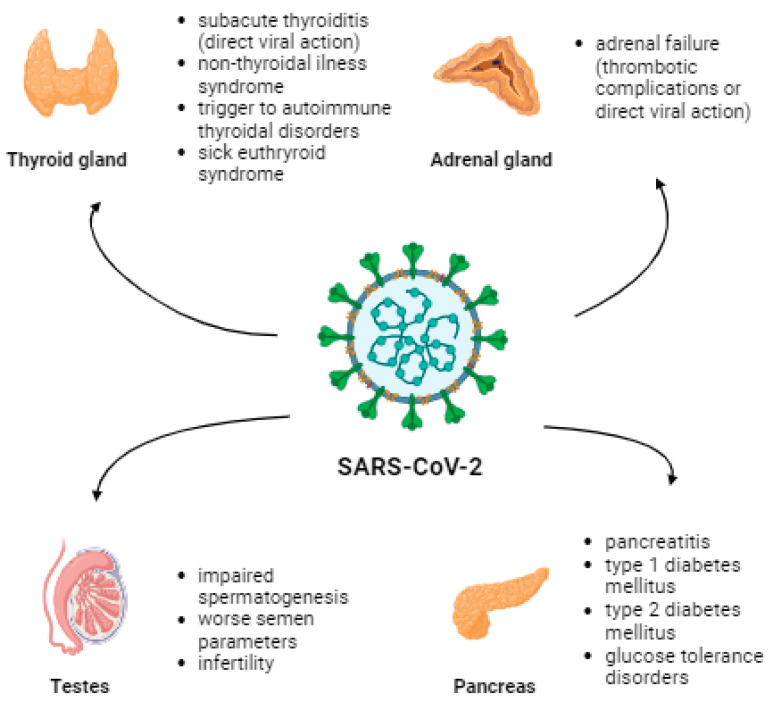
The effect of SARS-CoV-2 infection on certain endocrinologic and metabolic complications associated with the presence of the ACE2 receptor.

Elderly males and people with underlying diseases such as diabetes, chronic heart, and chronic lung diseases are more susceptible to COVID-19 infection and its complications [14,15]. ACE2 receptors are present in the lungs and the thyroid, adrenal glands, testes, pancreas, and ovaries [16]. Therefore, the pathomechanism of endocrine dysfunction is due to direct destruction caused by a viral infection, epithelial dysfunction caused by a viral infection, immune response caused by uncontrolled secretion of pro-inflammatory cytokines, or disruption of the renin-angiotensin-aldosterone system (RAAS) [17]. The most common thyroid complications include subacute thyroiditis (SAT) and non-thyroidal illness syndrome (NTIS) [18]. Viral infection or thrombotic complications can lead to failure in the adrenal glands. Pancreatic complications may manifest as pancreatitis, T1D, T2D, or other glucose tolerance disorders [18]. Moreover, diabetes increases the risk of morbidity, severe COVID-19, and mortality [19]. An inflammatory process can occur in the testes, leading to impaired spermatogenesis and worsening semen parameters, affecting fertility [18]. Figure 1 presents the impact of SARS-CoV-2 infection on mentioned endocrinological and metabolic parameters.

Limited data are available regarding SARS-CoV-2 vaccination efficiency with metabolic disorders, especially obesity, T1D, and T2D. Therefore, this review aimed to summarize the current knowledge regarding whether and how metabolic disorders may affect the effectiveness of COVID-19 vaccines.

## 2. Insight into the Most Common Types of COVID-19 Vaccines

One hundred twenty-four vaccine candidates were being developed by 1 June 2020 and listed in the landscape summary of the World Health Organization (WHO) [20]. As of June 2021, 19 vaccines gained regulatory approval in at least one country [21], and six were given Emergency Use Listing (EUL) by the WHO [20]. Vaccines globally in use include messenger RNA (mRNA), viral vector, inactivated, and protein subunit vaccines [21]. Vaccines can, and often should, be mixed and matched [22]. The combination of mRNA and vector vaccines against SARS-CoV-2 (heterological vaccination schedule) has been shown to generate adequate antibody levels and a better cellular response than the repeated (homological) use of the same vaccine [23]. These conclusions apply to both the primary and booster schedules. The summary of available and best-described vaccines is presented in Table 1 and discussed below.

### 2.1. Mechanisms of Vaccine Activity

The model of mRNA vaccine presents a modern approach where genetically modified RNA and DNA prompt an immune response [24] and has attracted substantial scientific interest, especially in the COVID-19 aspect. Most mRNA vaccines utilize lipid nanoparticles (LNPs) as a tool for transfection [25]. LNPs support mRNA molecules and, together with helper lipids such as dioleoylphosphatidylethanolamine (DOPE), phosphatidylcholine, or cholesterol, contributing to stability, delivering efficiency of LNP or release of mRNA [26], form a complex of lipidized polymers which alters surface features of particles [27]. After the vaccine’s administration, their components are phagocyted by human cells, which leads to an endosome punctured by RNA condensing lipids, leading to a release of mRNA molecules into the cell’s cystosole, where protein synthesis occurs [27]. Two main types of RNA are currently being studied as vaccines: self-amplified RNA of viral origin and non-replicating mRNA [28]. Self-amplifying RNAs are typically the antigen and machinery for viral replication, whereas vaccines based on mRNA only encode the particular antigen with a translation of regions at 5 and 3’ [28]. Quick development, manufacturing, and administration are the most crucial advantages of mRNA-based vaccines compared to traditional ones [28]. Additionally, mRNA is not an infectious and integrating platform; i.e., the probability of potential infection or insertional mutagenesis is very low [28]. Moreover, mRNA can encode multiple antigens allowing their administration in a single vaccine [29], which is essential because administering a single dose enables vaccination against various diseases [30]. Typical disadvantages of mRNA vaccines include instability of mRNA and extremely low storage temperatures, which poses a substantial challenge to low- and medium-income countries [31].

The authorization of two mRNA vaccines—the BNT162b2 Comirnaty vaccine (Pfizer-BioNTech) [32] and the mRNA-1273 Spikevax vaccine (Moderna) [33] was announced at the start of 2021 and was very important in the response to the pandemic [33]. Pfizer-BioNTech is a nucleoside-modified (modRNA) vaccine and encodes perfusion-stabilized membrane-anchored SARS-CoV-2 full-length spike (S) protein [34]. Pfizer-BioNTech stimulates the production of anti-S protein IgG, whereas the response of T cells is yet to be explained [35]. The Moderna vaccine also encodes the S protein of SARS-CoV-2 and uses similar vaccine technology as Pfizer-BioNTech [31]. Table 1 presents a summary of the latest knowledge regarding mRNA vaccines against SARS-CoV-2.

AstraZeneca (AZD1222) and Johnson & Johnson (Ad26.COV2.S) are viral vector vaccines that use genetically engineered viral vectors to deliver genetic material into cells [36]. Viral vector vaccines work through the induction of T cells and humoral response [37]. Engineered DNA is introduced to a cell by a viral infection and is used to create an mRNA molecule. These mRNA particles form a template for S protein synthesis involved in SARS-CoV-2 infection. Once the protein is synthesized, the mRNA is dissolved and removed from the organism. The vector does not typically interact with mRNA and DNA, and what is more, used vectors are mild or harmless to human viruses, such as a chimpanzee adenovirus used with the AZD1222 vaccine. Upon coming into contact with a virus, the immune system of a vaccinated person would detect the foreign protein and create virucidal antibodies. The adenovirus vector utilized in AZD1222 belongs to a single non-replicating chimpanzee adenovirus vaccine vector called ChAdOx1, encoding full-length spike protein of SARS-CoV-2, previously used in vaccinations against MERS [38]. The use of ChAdOx1 was dictated by strong immunogenicity and a high probability of destruction by the immune system using other types of adenoviruses [39]. Until June 2021, AZD1222 was approved in 161 countries and is listed on EUL by the WHO [21]. Ad26.COV2.S is a monovalent vaccine that utilizes an adenovirus type 26 vector, which encodes the full-length SARS-CoV-2 spike protein from the Wuhan-Hu-1 isolate [29]. By June 2021, Ad26.COV2.S was approved in 52 countries and listed on EUL by the WHO [21]. A detailed summary of Pfizer-BioNTech and Moderna mRNA vaccines, as well as AstraZeneca and Johnson & Johnson viral vector vaccines can be found in Table 1.

**Table 1:** The summary of different types of vaccine action, mechanisms involved in response to vaccination, effectiveness, and complications.

Vaccine Name	Recommended Dosage Regimen	Antibody Type	Mechanism of Immune Stimulation	Vaccination Effectiveness against Disease and Complications	References
Pfizer-BioNTech (BNT162b2)	Two doses 4–8 weeks apart, intramuscularly administered for individuals [40]	IgA, IgG [41]	mRNA lipid nanoparticle containing a superficial spike protein of SARS-CoV-2 virus -> intramuscular injection -> host cell binding -> mRNA insertion into cytoplasm -> synthesis of the viral spike proteins (translation) -> two types of protein evolution: MHC-2 (antigen-presenting cells (APC)) and MHC-1 (all nucleated cells) complex -> activation of APC and attraction of the immune cells, particularly CD4+ T helper cells (Th) -> binding of a viral spike protein by TCR membrane protein of Th cells and interaction of CD4 proteins with MHC-2 -> activation of Th cells and production of interleukins (Il) Il-2, -4, -5 -> differentiation of B-cells into plasma cells -> production of antibodies against the viral spike protein [31] At the same time, there is stimulation of Th cells to proliferate to T memory cells [31] CD8+ T-cell response: CD8+ T cells, also known as cytotoxic T lymphocytes -> recognition of viral spike protein fragments presented on MHC-I molecules -> interaction between the TCR on CD8+ T cells and the viral antigen-MHC-I complex, along with co-stimulatory signals -> activation of CD8+ T cells [31]	95% for disease, 87.5% for a severe course of COVID-19 [31]	[31]
Moderna (mRNA1273)	Two doses 8 weeks apart [42]	IgG [41]	Moderna and Pfizer-BioNTech exhibit the same mechanism of immune stimulation: they share the same amino acid sequence and encode the same S-2P protein. They differ in 5′-UTR and 3′-UTR designs and codon optimizations [43]. Stimulation of Th cells to proliferate to T memory cells [42].	94% for COVID-19, 100% for the severe course of the disease [31]	[31]
Astra-Zeneca Oxford AZD1222	Two doses 8–12 weeks apart [44]	IgG [45]	A modified chimpanzee DNA adenovirus containing DNA gene of SARS-CoV-2 spike protein -> intramuscular injection of the vaccine -> latching to host cells -> release of DNA into cytoplasm without incorporation into cellular DNA -> conversion into mRNA through enzymes of host cells -> translation -> T-cell activation (CD4, CD8) and production of antibodies [31] CD8+ T-cell response: recognition of viral spike protein fragments displayed on MHC-I molecules -> interaction between TCR on CD8+ T-cells along with presence of co-stimulatory signals -> multiplication and differentiation into cytotoxic T-cells able to kill viral infected cells [31]	About 65% following 1 dose, about 70% following 2 doses [31]	[31]
Janssen Johnson & Johnson (Ad26.COV2.S)	Two doses 2–6 months apart according to the WHO, 1 dose according to EUL [46]	anti-RBD IgG [47]	Ad26.COV2.S utilizes a similar mechanism of action to AZD1222 with the main difference of using Human Adenovirus serotype 26 containing the gene of SARS-CoV-2 spike protein [48].	About 72% in the United States of America, about 66% in Latin America, about 57% in South Africa [31]	[31]

### 2.2. Adaptive Immune Response—Cellular and Humoral

The adaptive immune system is a complex network of cells and molecules that plays a crucial role in defending the body against pathogens [49]. It consists of various components, including T lymphocytes (T cells), B lymphocytes (B cells), and antibodies [49]. T cells are a type of lymphocyte that is responsible for cell-mediated immunity [49]. They play a central role in coordinating and executing immune responses [49]. There are different types of T cells, but two key types involved in the adaptive immune response are cytotoxic T cells and T helper cells [49].

Cytotoxic T cells, also known as CD8+ T cells, are specialized in identifying and eliminating cells that have been infected by viruses or other intracellular pathogens [50]. They recognize specific antigens presented on the surface of infected cells and initiate a response to destroy them, thereby preventing the spread of the infection [50]. The presence of virus-specific CD8+ T cells is associated with better COVID-19 outcomes [49].

T helper cells, also known as CD4+ T cells, are essential for orchestrating immune responses [51]. They have the ability to recognize antigens presented by antigen-presenting cells (APCs) such as macrophages [52]. Once activated, T helper cells release chemical signals called cytokines, which stimulate other immune cells, including macrophages, cytotoxic T cells, and B cells [51]. T helper cells are particularly important for activating B cells and supporting their antibody production [51]. CD4+ T cells reduce the severity of COVID-19. The rapid induction of T helper cells in the acute course is associated with mild disease and accelerated virus clearance. Reduced or absent CD4+ T cells increase the risk of severe complications [49].

B cells are another type of lymphocyte that plays a critical role in the adaptive immune response [50]. They are responsible for humoral immunity, which involves the production of antibodies [50]. When B cells encounter an antigen that matches their specific receptors, they become activated [50]. T helper cells provide the necessary signals to stimulate B cells, leading to their proliferation and differentiation into antibody-secreting plasma cells [49]. Antibodies, also known as immunoglobulins, are proteins that can recognize and bind to specific antigens, marking them for destruction or neutralizing their effects [51].

The adaptive immune response is crucial for long-term protection against pathogens [49]. After an infection or vaccination, memory T cells and memory B cells are generated [49]. These memory cells “remember” the specific antigens encountered and allow for a faster and more effective immune response upon subsequent encounters with the same pathogen [49]. This is the basis for the long-term immunity conferred by both natural infections and vaccinations [49].

It is worth noting that while the primary focus of this response is on T cells, B cells, and antibodies, other cell types also contribute to the adaptive immune response [50]. Monocytes, for example, are a type of white blood cell that plays a role in inflammatory responses [50]. After vaccination, the presence of high levels of monocytes in the body indicates their participation in the immune response generated by the vaccine [50]. Their activation and recruitment to the site of infection or vaccination help initiate and coordinate the immune response [50].

### 2.3. Neutralizing Antibodies in COVID-19 Infection

Most COVID-19 infections involve the creation of neutralizing antibodies (Nabs) that block the entry and replication of a virus [52]. Nabs activity during infection and vaccination increases future immunity [53]. The primary Nab types involved in the immune response include IgA, IgG, and IgM [53]. Tests investigating seroreactivity with SARS-CoV-2 typically measure IgG, IgM, and total Ig antibody levels [54]. IgM levels peak early and quickly decay [54]. IgG levels peak slightly later and decay more slowly, remaining detectable for at least 4 months [55]. Critically ill patients generate more IgM and IgG antibodies against S and N proteins and peak IgM levels later than their mildly ill counterparts [56]. However, IgA antibodies dominate the immune response over other antigens, including IgM and IgG—as measured by number concentration. IgA antibody levels shortly grow upon first symptoms and peak in week three [57]. IgA measurements also have better sensitivity and specificity than IgG and IgM in hospitalized patients [57].

Nabs are generated during most infections and destroy SARS-CoV-2 viruses mainly via the renin-angiotensin (RAS) molecular pathway [53]. The RAS pathway involves an angiotensin-converting enzyme (ACE) and ACE2 [53]. ACE2 is a principal regulator of the RAS pathway, which is found in human cells [53]. Nabs block the SARS-CoV-2 virus from binding with a specific surface receptor of ACE2 by targeting the receptor-binding domain (RBD) [58]. RBD is located on the S1 subunit of the SARS-CoV-2’s S protein and binds with ACE2, which allows the SARS-CoV-2 virus to skip the cellular boundary, enter the human cell, and replicate [58]. Vaccines use the capability of Nabs to block the binding of the virus with the ACE2 receptor and subsequent entry to the cell [59]. Higher levels of ACE2 correspond to early-stage SARS-CoV-2 infection, and more rapid subsequent decays are associated with severe disease [60]. ACE2 also impacts adaptive immunity by activating immune system cells and increasing the production of IL-6, TNF-α, and other inflammatory cytokines [53].

### 2.4. Immune Response in Recovered and Vaccinated Patients

How vaccine- and recovery-induced immunities compare has not been established because both are based on active immunity that generates antibodies through humoral response and immunity cells through cellular response [61]. It has been shown that higher antibody concentrations come from vaccination than from recovery [61]. Average IgG, IgA, and Nab levels were higher for recovered vaccinated patients after the first dose than for non-recovered vaccinated patients. Still, while the IgG level was similar for both groups, IgA and Nab levels only increased for non-recovered patients [62]. Another study shows that antibody response upon the first dose of the Pfizer-BioNTech vaccine was 6.8 times higher in recovered than non-recovered patients [61]. Similar antibody levels were found for second-dose non-recovered patients and first-dose recoverees with Pfizer-BioNTech [55]. Additionally, significantly lower antibody levels for second-dose non-recovered patients than for first-dose recoverees with Pfizer-BioNTech were also observed, which indicates a more robust humoral response in recovered patients upon the first vaccine dose than that for unrecovered patients that underwent two courses of vaccination [63]. Those findings are mentioned in detail in Table 1.

### 2.5. How to Measure Vaccine Response

Vaccine response can be measured using numerous serological tests, which mostly measure the anti-Receptor Binding Domain (RBD) or ACE2 antibodies [64,65].

Enzyme-Linked Immunosorbent Assay (ELISA) is widely used to measure vaccine-induced antibody responses [66]. They detect and quantify antibodies, often targeting the anti-Receptor Binding Domain (RBD) or ACE2 antibodies specific to SARS-CoV-2 [66]. ELISA assays provide indirect measurements of T-cell memory activity, complementing the assessment of humoral immune responses [66].

Enzyme-Linked Immunospot Assay (ELISPOT) is a technique that allows the detection and quantification of individual immune cells secreting specific cytokines, such as interferon-gamma (IFN-γ) [67]. It provides a means by which to assess cellular immune responses following vaccination [67]. In an ELISPOT assay, immune cells, such as T cells, are isolated from blood samples and stimulated with SARS-CoV-2-specific peptides [67]. The cells are then plated on an ELISPOT plate, and cytokine-producing cells, indicated by spots, are detected and counted. ELISPOT provides valuable information about the number and functionality of vaccine-induced T cells [67].

Flow cytometry is a powerful technique used to analyze individual cells in a heterogeneous population [68]. In the context of vaccine response, flow cytometry can be utilized to assess the production of specific cytokines, such as IFN-γ, by immune cells [68]. Intracellular cytokine staining (ICS) is a flow cytometry-based method that involves the detection of cytokines within cells [68]. Antigen-specific cytokine secretion assay (AIM) is another flow cytometry-based technique that evaluates the production of cytokines by immune cells following antigen stimulation [68]. These flow cytometry methods provide detailed information about the phenotype and functional properties of vaccine-induced immune cells [68].

The IGRA test consists of two steps: in vitro or ex vivo stimulation of T-lymphocytes (the sample is heparinized whole blood) with SARS-CoV-2-specific peptides [69]. It induces IFN-γ production due to prior contact with the pathogen [69]. IFN-γ levels are measured in plasma extracted from tubes after incubation and centrifugation [69]. Resulting IFN-γ measurements have been linked to cellular responses to SARS-CoV-2 exposure [69]. High levels (above 25 IU/L) are associated with evidence of T-lymphocyte activity upon SARS-CoV-2 antigen exposure [69].

## 3. Obesity and Vaccines

Obesity is a cause of metabolic disturbances, which result in the activation of the immune system, indicated by the elevation of inflammatory plasma biomarkers [70]. In addition, lymphoid tissue undergoes architectural and integral alterations, shifting toward inflammatory phenotypes in the leukocyte population in obesity [70]. Seroconversion, producing specific antibodies in response to infection or vaccination, was also impaired among obese individuals [71]. Unfortunately, these factors are connected with poor vaccine response and complications of infectious diseases, which show a significant impact of obesity [70].

In COVID-19 vaccine studies, a lot of attention has been focused on vaccination-dependent responses in obese patients [72,73,74,75]. Both the mRNA vaccine and the vector vaccine were shown to induce virus-neutralizing antibodies and T-cell immune responses in obese patients [76], which is connected with the fact that the efficacy of COVID-19 vaccination largely depends on memory T cells [76]. This response has been shown to be impaired in obesity, suggesting that vaccines may be less effective in obese individuals [76]. Another study noticed a strong correlation between BMI class and antibody titers, and the humoral response was more effective in underweight and average weight than in overweight and obese individuals [74]. Another clinical study has shown that all approved COVID-19 vaccines effectively protect the obese in the short term [77]. However, based on the available literature, there is no data on how long the protective period persists in the obese population. Interestingly, obesity was characterized by an impaired memory B cell response, which reduced long-term protection against reinfection [77]. Furthermore, obesity can reduce the long-term efficacy of COVID-19 vaccination by altering the expression of programmed death receptor 1 (PD1) and programmed death ligand (PD-L1) on effector cells (lim Te), which causes an impaired stem cell response [77]. Moreover, obesity can cause increased production of reactive oxygen species (ROS), causing a shortening of the telomeres of immune cells and, as a result, reducing the proliferation of lim Te and memory B lymphocyte populations [77]. A summary of obesity’s effect on the immune response to SARS-CoV-2 vaccination is presented in Figure 1.

So far, the results of studies on the effectiveness of vaccines in obese people are still uncertain since adipose tissue, as an endocrine organ itself, modulates innate and acquired immune responses. Research on the SARS-CoV-2 vaccine should be developed and closely monitored, especially among obese populations. Table 2 summarizes the effect of obesity on vaccine response in different types of vaccines.

### Insulin Resistance (IR) and Antibody Response following COVID-19 Vaccinations

Insulin resistance (IR) is defined as reducing tissue’s sensitivity to insulin, resulting in insufficient insulin secretion in the pancreas to regulate blood glucose levels [82]. IR leads to the impairment of glucose regulation and is essential in cardiovascular disease development and diabetes [83]. IR is a global problem affecting approximately 50% of the world’s adult population [82].

Interestingly, some research has indicated the contribution of SARS-CoV-2 to the development of IR via integrated stress response (ISR) [84]. ISR leading to the activation of serine/threonine protein kinases: PKR-like endoplasmic reticulum kinase (PERK), PERK double-stranded RNA-dependent protein kinase (PKR), heme-regulated eukaryotic translation factor 2a (eIF2a), and general control non-derepressible 2-kinase (GCN2), which phosphorylate elF2a on serine resulting in the translation of particular genes crucial in the survival of cells [85]. The activation of a minimum of two serine/threonine kinases through stress factors results in the downregulation of the insulin signaling pathway because of the phosphorylation of serine, a substrate of the insulin receptor, which impairs insulin activity [86]. It has also been suggested that RNA fragments of SARS-CoV-2 can activate PRK, which generates phosphorylation of insulin receptor substrate 1 (IRS-1) serine causing insulin resistance [87]. Moreover, a “cytokine storm” during COVID-19 or a hormonal imbalance of cortisol can activate these kinases participating in the development of IR [88]. However, the exact molecular mechanism is still under investigation.

Individuals with IR are at high risk of severe SARS-CoV-2 infection, as patients with preexisting IR also often have other comorbidities responsible for the increased severity of the disease, including diabetes mellitus, hypertension, and hyperglycemia [89]. Coexisting obesity among IR individuals is also a risk factor for lung injury [90], and the presence of IR in diabetes mellitus has been found to cause hyperglycemia and chronic inflammation resulting in dysfunction of the lungs [84]. A potential explanation of increased morbidity among IR individuals seems to be linked with the expression of ACE2, which converts angiotensin II (AngII) into angiotensin 1–7 (Ang 1–7), which regulates blood pressure and results in the decrease of IR [91]. Viral binding of ACE2 increases the concentration of AngII and exaggerates its activity, which leads to inflammation, hypertension, and cardiac dysfunction, increasing the severity of COVID-19 [92]. However, there is no data regarding specific symptoms of COVID-19 among IR individuals. Additionally, Joo et al. showed a loss of antibodies against hepatitis B in IR patients compared to non-diabetic subjects [93]. These results suggest that IR patients can also respond differently to other vaccines, but limited data regarding vaccinations against SARS-CoV-2 in patients exclusively with IR do not provide this information. Moreover, IR is associated with poor metabolic health conditions (e.g., obesity or diabetes) [94] and should be considered an essential factor of impaired immunogenicity following vaccination, as the effectiveness of vaccinations against SARS-CoV-2 is lower among individuals with chronic metabolic conditions [95]. The summary of the IR effect on the immune response to vaccination against SARS-CoV-2 is presented in Figure 1.

## 4. T1D and Immunogenicity of SARS-CoV-2 Vaccinations

Type 1 diabetes mellitus (T1D) is a chronic autoimmune disease in which insulin-producing β cells in the pancreas are destroyed by their autoimmune reaction driven by autoimmune T-cells [96]. At the beginning of the COVID-19 pandemic, T1D patients were poorly represented among diabetes patients [97,98], probably due to the younger age of the population of potential patients [99] or precautions, as the majority of these individuals are aware of the disease [100]. Two years after the start of the pandemic of SARS-CoV-2, studies have indicated that T1D individuals are at high risk of a severe course of COVID-19 [101,102,103,104], with even a three-fold higher risk of mortality, admission to intensive care units, and mechanical ventilation in comparison to type 2 diabetes mellitus (T2D) individuals [101]. Moreover, a recent study also revealed increased SARS-CoV-2 seropositivity among T1D patients compared to healthy controls, predisposing them to severe SARS-CoV-2 infection [105]. To the best of our knowledge, no research has indicated the differences between COVID-19 symptoms in T1D subjects and healthy individuals.

**Figure 2:**
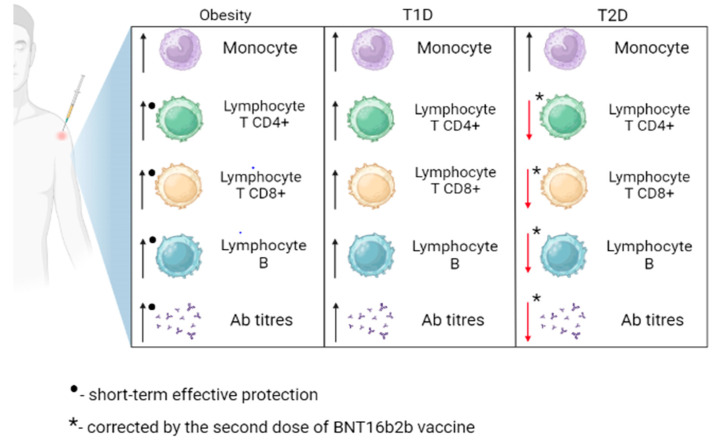
Immune response to SARS-CoV-2 vaccination in obese, type 1 diabetes (T1D), and type 2 diabetes (T2D) patients. Ab, antibodies. Obesity is associated with a transient increase in CD4+ T lymphocytes, CD8+ T lymphocytes, B lymphocytes, and antibody levels. The response to vaccination in individuals with T1D is comparable to that of healthy individuals. The second dose of the BNT16b2b vaccine led to an improvement in the levels of CD4+ T lymphocytes, CD8+ T lymphocytes, B lymphocytes, and antibody levels in individuals with T2D.

The high risk of severe COVID-19 results from impaired cellular response among T1D individuals, with the decreased release of T-cell-specific factors, impairing the inactivation of the virus [106]. Alterations of humoral response were not found, as there was no significant difference in the level of anti-SARS-CoV-2 antibodies between T1D and subjects without diabetes [106,107]. In addition, the immune response to COVID-19 vaccination in people with diabetes mellitus COVAC-DM revealed that antibody response among T1D subjects following the third dose of COVID-19 vaccination is comparable to healthy participants [108]. Data presenting glycemic control’s impact on immune response after vaccination indicates no association between humoral immune response and glycemic control among T1D individuals [107]. However, further research should be conducted to fully prove the presented results’ consistency. The mentioned changes caused by T1D are shown in Figure 2.

### T2D and Immune Response to SARS-CoV-2 Vaccinations

T2D is the most common type of diabetes, accounting for approximately 85–90% of diabetes cases [109]. It is caused by defective insulin secretion by pancreatic β-cells and the inability of insulin-sensitive tissues to appropriately respond to insulin [110]. Diabetes is a comorbidity associated with morbidity and mortality of COVID-19 [111]. People with T2D have an increased risk of severe course of COVID-19 in comparison to people without diabetes [112,113]. Higher hospitalization rates, in-hospital and total deaths are also more frequently observed in seropositive people with diabetes [114]. However, patients with diabetes and better-controlled blood sugar have a lower severity of COVID-19 pneumonia and a lower risk of death than patients with poorly controlled blood sugar during hospitalization [80].

People with diabetes have compromised innate and adaptive immune systems [115,116]. T cells have been reported to be abnormally differentiated in individuals with T2D [116]. Patients with hyperglycemia and IR have a reduced number of circulating helper T cells, increased senescent T cells, impaired T-cell migration, and decreased T-cell lysis [109]. Any abnormality in T-cell quantity or function is likely to damage B-cell activation and the subsequent production of neutralizing antibodies [111,116,117]. It also reduces protective T- and B-cell responses against viral pathogens during a SARS-CoV-2 infection [109]. Moreover, patients with T2D are more likely to have non-detectable anti-SARS-CoV-2 antibodies following initial infection than those without T2D, even after two weeks [116,117]—which may put T2D patients at higher risk from COVID-19. T2D cases have indicated reduced Th1/Th2 cytokines ratios and the numbers of CD4+, and CD8+ cells, compared with non-diabetic people with SARS-CoV-2 infections [118]. There is, however, inconsistent evidence as to whether T2D impairs seroconversion following COVID-19 vaccination. It has been shown that antibody response and seropositivity were lower in T2D patients one to four weeks after the total dose of COVID-19 vaccination than in healthy patient groups [115,116]. Both T2D and non-diabetics show a robust immune response to vaccination, as demonstrated by high antibody titers. However, SARS-CoV-2 IgG and neutralizing antibody titers were lower in T2D patients [119]. In summary, the BNT162b2 vaccine induced CD4+, CD8+ T cells, IgGs, and neutralizing antibodies in T2D and non-diabetic patients.

Nevertheless, the CD4+ cellular response in T2D patients was found to be defective shortly before the second shot, as measured by fewer CD4+ cells. This initial defect was corrected by the second dose of BNT16b2b, resulting in comparable levels of CD4+, CD8+ T cells, IgGs, and neutralizing antibodies at 3–6 months after vaccination [81]. Interestingly, T2D patients with HbA1c > 7% showed a significant decrease in virus-neutralizing antibodies compared to patients with average blood glucose and T2D patients with good blood glucose control [120,121]. Furthermore, the presence of diabetes and hyperglycemia does not affect the kinetics or persistence of the neutralizing antibody response [80,122].

To date, all mentioned changes caused by T2D in response to SARS-CoV-2 infection are presented in Figure 2 and Table 3. Research on the association between T2D and the severity of COVID-19 is ongoing; therefore, the detailed effect of T2D on vaccination response requires further study.

## 5. Conclusions

This work summarizes knowledge regarding the mechanisms of action of the most popular vaccines against COVID-19 and the immune response in patients with obesity and glucose disruptions such as IR, T1D, and T2D. The effects of metabolic disorders on immune response after vaccination are summarized in Figure 1. The current knowledge does not allow for an unequivocal statement on how T1D affects the vaccination effect and how T1D modulates the immune response. Recent research has indicated an impaired response to vaccination in T2D patients primarily due to the impairment of T-cells and related activation of B cells, which are responsible for synthesizing antibodies. The vaccination of T2D patients is recommended according to the current vaccination schedules for people at increased risk of a severe course of COVID-19. Data presented in the review does not support any clear recommendation of a specific vaccine for populations with IR, obesity, or diabetes. Unfortunately, almost four years after the first cases of SARS-CoV-2 infection and three years after the first vaccine, the effect of metabolic disorders such as obesity, IR, and diabetes on COVID-19 vaccination is still not well known, even though a few types of vaccine as a mechanism to protect against virus infection were developed, data are inconsistent, and debate is still ongoing.

## Figures and Tables

**Table 2:** Summary of the effect of obesity on vaccine response in different types of vaccines.

Vaccine Name	Immune Response in Diabetes Mellitus and Other Related Diseases	References
Pfizer-BioNTech (BNT162b2)	Association between metabolic syndrome and weaker immune response [78] Improvement of humoral response in underweight and normal-weight individuals in comparison to obese [74] No association between BMI and antibody response [79]	[74,78,79]
Moderna (mRNA1273)	No significant differences in the efficacy of the Moderna vaccine among the population with obesity compared to people without obesity [72]	[72]
Astra-Zeneca Oxford AZD1222	Acute hyperglycemia 20–36 days after administration of the first dose vaccine in obese dyslipidemic male patients (two with prediabetes) [80]	[80]
Janssen Johnson & Johnson (Ad26.COV2.S)	No significant difference in the efficacy in obese individuals compared to normal-weight subjects [81]	[81]

**Table 3:** Vaccine response in T2D by vaccine types.

Vaccine Name.	Immune Response in Diabetes Mellitus and Other Related Diseases	References
Pfizer-BioNTech (BNT162b2)	Association between metabolic syndrome and weaker immune response [78] Association between diabetes and impaired fasting glycemia (IFG) following vaccination [123] Impaired antibody response among diabetic patients [124]	[78,123,124]
Moderna (mRNA1273)	High level of safety and efficacy in high-risk subgroups (obesity, severe obesity) after vaccination in preliminary data [72]	[72]
Astra-Zeneca Oxford AZD1222	Acute hyperglycemia 20–36 days after administration of the first dose vaccine in obese dyslipidemic male patients (two with prediabetes) [124] Diabetic individuals presented post-vaccination hyperglycemia 1–6 days after the first dose [124]	[124]

## Data Availability

No new data were created.
